# Comparative Analysis on the Key Enzymes of the Glycerol Cycle Metabolic Pathway in *Dunaliella salina* under Osmotic Stresses

**DOI:** 10.1371/journal.pone.0037578

**Published:** 2012-06-04

**Authors:** Hui Chen, Yan Lu, Jian-Guo Jiang

**Affiliations:** 1 College of Food Bioengineering, South China University of Technology, Guangzhou, China; 2 Institute of Hydrobiology, Chinese Academy of Sciences, Wuhan, China; 3 School of Biological Science and Engineering, South China University of Technology, Guangzhou, China; Mayo Clinic, United States of America

## Abstract

The glycerol metabolic pathway is a special cycle way; glycerol-3-phosphate dehydrogenase (G3pdh), glycerol-3-phosphate phosphatase (G3pp), dihydroxyacetone reductase (Dhar), and dihydroxyacetone kinase (Dhak) are the key enzymes around the pathway. Glycerol is an important osmolyte for *Dunaliella salina* to resist osmotic stress. In this study, comparative activities of the four enzymes in *D. salina* and their activity changes under various salt stresses were investigated, from which glycerol metabolic flow direction in the glycerol metabolic pathway was estimated. Results showed that the salinity changes had different effects on the enzymes activities. NaCl could stimulate the activities of all the four enzymes in various degrees when *D. salina* was grown under continuous salt stress. When treated by hyperosmotic or hypoosmotic shock, only the activity of G3pdh in *D. salina* was significantly stimulated. It was speculated that, under osmotic stresses, the emergency response of the cycle pathway in *D. salina* was driven by G3pdh via its response to the osmotic stress. Subsequently, with the changes of salinity, other three enzymes started to respond to osmotic stress. Dhar played a role of balancing the cycle metabolic pathway by its forward and backward reactions. Through synergy, the four enzymes worked together for the effective flow of the cycle metabolic pathways to maintain the glycerol requirements of cells in order to adapt to osmotic stress environments.

## Introduction

*Dunaliella salina*, an extremely halotolerant, unicellular, green, and motile algae, is one member of genus *Dunaliella*, which is unique in its remarkable ability to survive in media containing a wide range of NaCl concentrations, ranging from about 0.05 M to saturation (around 5.5 M), while maintaining a relatively low intracellular sodium concentration in cells [1]. This remarkable osmotic adaptability is mediated primarily by the massive *de novo* synthesis of the osmolyte glycerol under salt stress [2]. In addition, *D. salina* is also one of the best sources of natural β-carotene, which could accumulate large amounts of β-carotene in cells under high salt stress [3]–[6].

**Figure 1:**
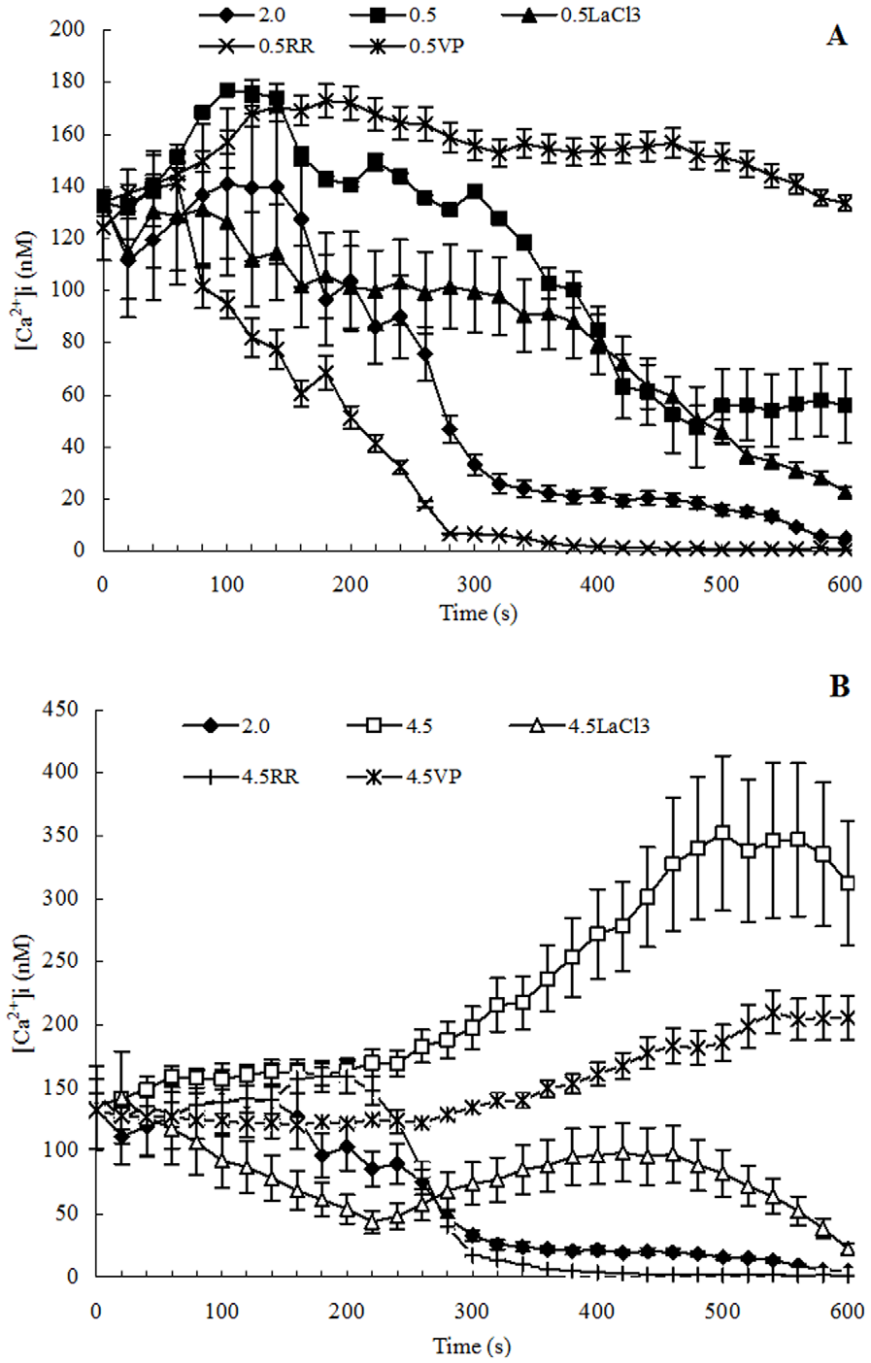
The pathway of glycerol metabolism in *Dunaliella*.

In *Dunaliella*, glycerol may be synthesized via two different metabolic pathways: one using a photosynthetic product and the other via the metabolic degradation of starch in cell [2], [7], [8]. Figure 1 shows the metabolism pathway of glycerol in *Dunaliella*. Firstly, via glycolysis pathway the glucose synthesized from photosynthesis or hydrolyzed from starch is converted to fructose-1, 6- bisphosphate and next to dihydroxyacetone phosphate (DHAP), which is converted to glycerol-3-phosphate by glycerol-3-phosphate dehydrogenase (G3pdh). Finally, glycerol-3-phosphate is converted to glycerol by glycerol-3-phosphate phosphatase (G3pp) [9], [10]. In the pathway of glycerol degradation, excess glycerol is removed by oxidation to dihydroxyacetone (DHA) catalyzed by glycerol dehydrogenase (also known as DHA reductase, Dhar), and then DHA is converted to DHAP catalyzed by DHA kinase (Dhak) [11]–[13].

In higher plants and algae, G3pdh is referred to as DHAP reductase, because at physiological pH and substrate, the enzyme is essentially inactive as a dehydrogenase [12]. The presence of DHAP reductase has been reported in *Dunaliella tertiolecta*, and three isoforms of DHAP reductase have been separated from *D. tertiolecta* [12], [14]. The chloroplastic osmoregulator DHAP reductase and glycerol-3-phosphate phosphatase both were stimulated by NaCl and have a rather sharp pH optimum around 7.0. It was reasonable to postulate that the osmoregulator form of DHAP reductase might be tightly associated with the glycerol-3-phosphate phosphatase in a complex, which might result in a direct conversion of DHAP to glycerol [15]. In addition, a research demonstrated the presence of an enzyme, Dhar, in cell-free extracts of *Dunaliella parva*, which catalyzes the reversible reaction between glycerol and DHA, and may play a major role in the osmotic regulation mechanism in *Dunaliella* [16].

According to Figure 1, four key enzymes, (NAD^+^)-dependent G3pdh, G3pp, NADPH-specific Dhar and Dhak catalyze a circular pathway in glycerol metabolisms, and some steps in this pathway are reversible. Given the characteristics of the metabolic pathways, in the present research, the correlations among the four enzymes in activities and activity changes were investigated in order to estimate the roles of the key enzymes in glycerol metabolic flow direction in *D. salina* under various salt stresses.

## Materials and Methods

### Continuous Cultivation of *D. salina* Under Salt Stresses

*D. salina* strain (FACHB-435) was obtained from Freshwater Algae Culture Collection of the Institute of Hydrobiology, Chinese Academic of Sciences. Cells of *D. salina* were cultivated in the culture medium containing 2.0 M NaCl at 26°C and 108 µmol m^-2^ s^-1^ provided by cool-white uorescent lamps, under a 14/10 h light/dark cycle with shaking at 96 rpm according to Chen et al. [17]. Cells of *D. salina* at log phase or late log phase were harvested by centrifugation at 5,000 g for 15 min at room temperature. Then the algal pellets were transferred respectively to fresh medium containing 0.5, 1.0, 1.5, 2.0, 2.5, 3.0, 3.5, 4.0, 4.5, and 5.0 M NaCl, and the inoculum concentration was 1% (w/v). Each culture was set three parallel samples. These cultures were cultivated at 26°C and 108 µmol m^-2^ s^-1^ provided by cool-white fluorescent lamps, under a 14/10 h light/dark cycle with shaking at 96 rpm. After cultivated for about 15 days, cells of *D. salina* at log phase in different salinity were harvested by centrifugation at 5,000 g for 15 min at 4°C for next experimental procedure.

### Hypoosmotic or Hyperosmotic Stress Culture

100 ml of *D. salina* culture containing 2.0 M NaCl was put into ten centrifuge tubes (10 ml/tube), and algae cells in these tubes were harvested by centrifugation at 5,000 g for 15 min at 4°C. Then algae pellets were respectively resuspended in isovolumetric fresh mediums, which contained 0.5, 1.0, 1.5, 2.0, 2.5, 3.0, 3.5, 4.0, 4.5 and 5.0 M NaCl, respectively. Each culture was set three parallel samples. After 2 h, resuspended algae cells were harvested by centrifugation at 5,000 g for 15 min at 4°C for next experimental procedure.

### Enzyme Extraction

The crude enzyme extracts were obtained according to the method of Chen et al. [8] with some modifications. The algal pellets collected by centrifugation were washed with enzyme extraction buffer (100 mM Tris, 20 mM ascorbic acid, pH 6.9), this process is repeated several times. The washed algal pellets were resuspended in 2 ml enzyme extraction buffer, and then ultrasonic treatment at 200 W for 5 min (5 s working time and 10 s interval in a cycle) in an ultrasonic cell disruptor at 4°C. The supernatants (crude enzyme extracts) were collected by centrifugation at 13,000 g for 30 min at 4°C. Samples were used for the following activities analysis of the four enzymes or diluted with glycerol by 50% (w/v) and placed at –20°C until analysis.

### (NAD^+^)-dependent G3pdh Activity

The activity of G3pdh, which catalyzed a reversible reaction, was analyzed according to the method of Wei et al. [18] with some modifications. The forward reaction mixture of 3 mL contained pH 6.9 buffer solution (33.3 mM Hepes, Tricine and Mes), 0.2 mM NADH, 1 mM DHAP and 200 µL of enzyme extract. The backward reaction mixture of 3 mL contained 50 mM glycine-NaOH buffer solution (pH 10), 250 mM glycerol-3-phosphate, 4 mM NAD+ and 200 µL of enzyme extract. The reaction mixture without enzyme extract served as control. 3 mL of deionized water was used as blank. G3pdh activity was assayed at 25°C after adding coenzyme and determined by spectrophotometer at 340 nm. G3pdh activity (U) is defined as the amount of enzyme that caused per micromoles NADH oxidation or per micromoles NAD+ reduction per minute. G3pdh activity is calculated:
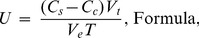
(1)


where Cs is the concentration of NADH produced or consumed in reaction mixture (nmol/mL); Cc is the concentration of NADH produced or consumed in control (nmol/mL); Vt is total reaction mixture volume (mL); Ve is enzyme extract volume in sample (mL); T is reaction time (min). Units of specific enzyme activity (U/mg) are expressed as micromoles per minute per milligram of protein.

A relationship curve of protein concentration (mg/mL) (*y*) against OD_595_ value (*x*) was plotted and the protein concentration was calculated according to the regression equation (1): *y* = 1.575*x*-0.0170, R^2^ = 0.9969. From the relationship curve between OD_340_ and NADH concentration regression equation (2): *Y* = 235.8*X*+0.0118, *R^2^* = 0.99002, where *Y* represents NADH concentration (nmol/mL) and *X* represents OD_340_ value, the NADH concentration was obtained by determining OD_340_.

### G3pp Activity

The activity of G3pp was analyzed according to the method described previously [19], [20] with modifications. The reaction mixture contained 20 mM tricine buffer (pH 7.0), 1 M glycerol-3-phosphate, 5 mM MgCl_2_, and 200 µL of extract in a final volume of 3 mL. The reaction mixture reacted at 37°C for 30 min, and the reaction was ended by adding 300 µL of 50% HClO_4_. Then the isometric vanadium molybdate reagent was added and the absorbance of mixture was determined by spectrophotometer at 415 nm. G3pp activity (*U*) is defined as the amount of enzyme that causes release per micromole inorganic phosphorus per minute at 37°C and pH 7.0. G3pp activity is calculated according to formula (1), here *C_s_* is the concentration of inorganic phosphorus produced in reaction mixture (nmol/mL); *C_c_* is the concentration of inorganic phosphorus produced in control (nmol/mL); *V_t_* is total reaction mixture volume (mL); *V_e_* is enzyme extract volume in sample (mL); *T* is reaction time (min). Units of specific enzyme activity (U/mg) are expressed as micromoles per minute per milligram of protein.

The protein concentration was calculated according to the regression equation (1). From the relationship curve between OD_415_ and inorganic phosphorus concentration regression equation (3): *y* = 1111*x*-4.222, *R^2^* = 0.9932, where *y* represents inorganic phosphorus concentration (nmol/mL) and *x* represents OD_415_ value, the inorganic phosphorus concentration was obtained by determining OD_415_.

### NADPH-specific Dhar Activity

The activity of Dhar, which catalyzed a reversible reaction, was analyzed according to the method of Ben-Amotz and Avron [16] and Ghoshal et al. [14]. The forward reaction mixture of 1 mL contained 30 mM tricine-glycine buffer (pH 9.2), 50 µM NADP^+^, 2.4 mM glycerol and 100 µL of enzyme extract. The backward reaction mixture of 1 mL contained 30 mM tricine buffer (pH 7.4), 100 µM NADPH, 5 mM DHA and 100 µL of enzyme extract. Dhar activity was assayed after adding NADP^+^ or DHA and determined by spectrophotometer at 340 nm. Dhar activity (*U*) is defined as the amount of enzyme that causes oxidation per micromole NADPH or reduction per micromole NADP^+^ per minute. Units of specific enzyme activity (U/mg) are expressed as micromoles per minute per milligram of protein.

The protein concentration was calculated according to the regression equation (1). From the relationship curve between OD_340_ and NADPH concentration regression equation (4): *y* = 250.0*x*-0.3250, *R^2^* = 0.9919, where *y* represents NADPH concentration (nmol/mL) and *x* represents OD_340_ value, the NADPH concentration was obtained by determining OD_340_.

### Dhak Activity

The activity of Dhak was analyzed according to the method of Johnson et al. [21] and Wang et al. [22] with some modifications. Dhak activity was followed at 30°C. The reaction mixture contained 1 mM DHA, 1 mM ATP, 1 mM MgCl, 0.1 mM NADH, 2 pg of G3pdh from rabbit muscle, 10 mM α,α-dipyridyl, 100 mM glycerol, 50 mM triethanolamine-hydrochloride buffer (pH 7.0), and 50 µL of extract in a final volume of 1 mL. Dhak activity is defined as the rate of ATP-dependent NADH oxidation, monitored by optical density at 340 nm. Units of specific enzyme activity (U/mg) are expressed as micromoles per minute per milligram of protein at 30°C.

The protein concentration was calculated according to the regression equation (1). The NADH concentration was obtained from regression equation (2).

### Statistical Analyses

Each result shown was the mean of three replicated studies. Statistical analysis of the data was performed using the program SPSS-13, and significance was determined at a 95% or 99% confidence limit.

## Results and Discussion

### Enzyme Activity

**Figure 2:**
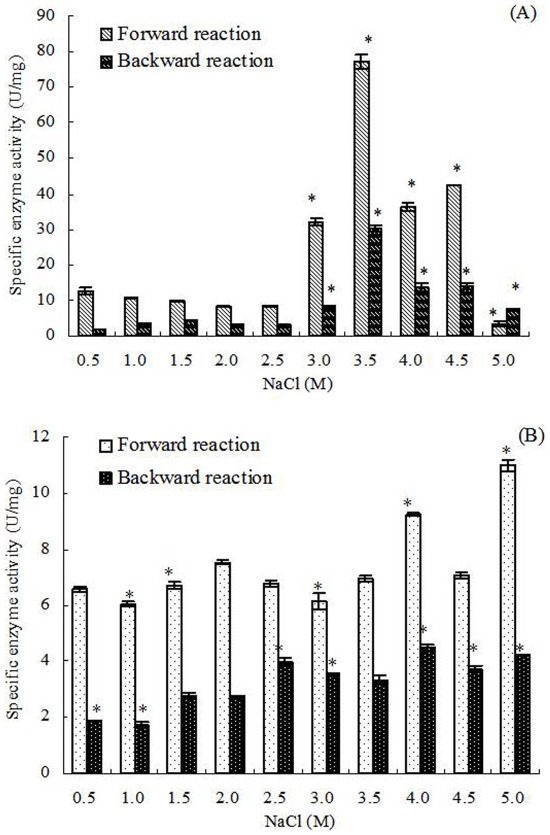
(NAD^+^)-dependent G3pdh activity in *D. salina* cells under different salinity stresses. (A): Cells grown chronically at various salinities; (B): Cells treated by hyperosmotic or hypoosmotic shock. Columns represent the means of three replicated studies in each sample, with the SD of the means (T test, P<0.01). The significance of the differences between the control (2.0) and test values were tested by using one-way ANOVA. *, P<0.05 vs control.

The changes of forward and backward reaction activities of G3pdh in *D. salina* grown continuously under 0.5–2.5 M NaCl were minor, but both increased significantly when salinities increased to 3.0 and 3.5 M NaCl (Figure 2). The activities of both reactions reached highest at 3.5 M NaCl (77.16±1.97 and 30.11±0.91 U/mg, respectively), and then decreased when salinities increased to 4.0–5.0 M NaCl. When algal culture of 2.0 M NaCl was treated by hypoosmotic or hyperosmotic shock, both forward and backward reaction activities of G3pdh showed the escalating trends, but the trends were unconspicuous and the activity changes were minor on the whole. Furthermore, relative to the significant increase in activity from 0–3.5 M NaCl in continuous cultivation, both forward and backward reaction activities of G3pdh in cells treated by hypoosmotic or hyperosmotic shock remained at lower levels, less than 10 U/mg on the whole. In addition, it was found that the forward reaction activities of G3pdh were always higher than the backward reaction activities at most of treatments (Figure 2).

**Figure 3:**
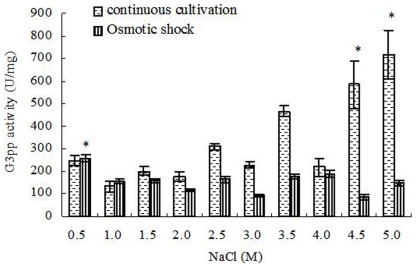
G3pp activity in *D. salina* cells under different salinity stresses. Columns represent the means of three replicated studies in each sample, with the SD of the means (T test, P<0.05). The significance of the differences between the control (2.0) and test values were tested by using one-way ANOVA. *, P<0.05 vs control.

In continuous cultivation, the activities of G3pp in *D. salina* increased significantly with increased salinities at different salinities, the maximum activity was detected under 5.0 M NaCl, which was 716.69±105.42 U/mg (Figure 3). However, when treated by hypoosmotic or hyperosmotic shock, the activities of G3pp in some treatments decreased with increased salinities. The activity in *D. salina* treated by 5.0 M NaCl (149.02±14.34 U/mg) was much lower than that of continuous cultivation under 5.0 M NaCl, while both the activities were almost equal at 0.5 M NaCl, which were 248.78±22.31 and 258.83±17.16 U/mg, respectively (Figure 3).

**Figure 4:**
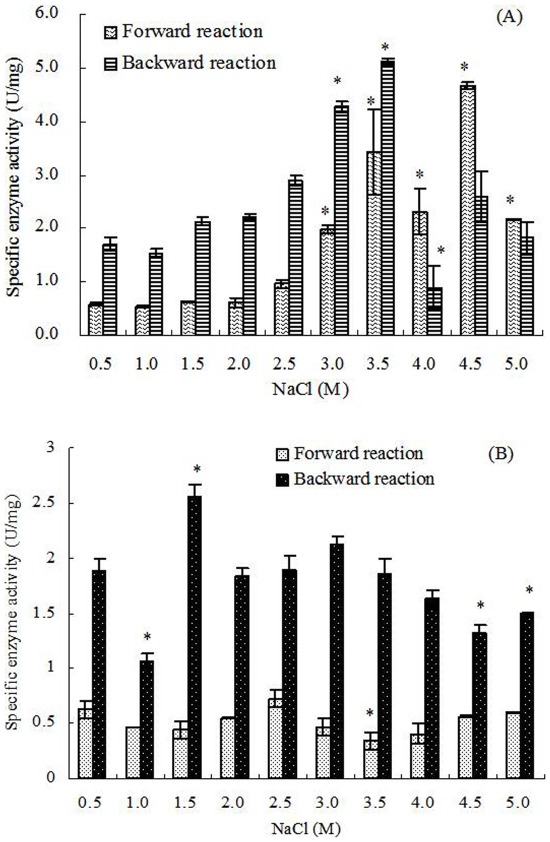
NADPH-specific Dhar activity in *D. salina* cells under different salinity stresses. (A): Cells grown chronically at various salinities; (B): Cells treated by hyperosmotic or hypoosmotic shock. Columns represent the means of three replicated studies in each sample, with the SD of the means (T test, P<0.05). The significance of the differences between the control (2.0) and test values were tested by using one-way ANOVA. *, P<0.05 vs control.

The forward reaction activities of Dhar increased relatively with increased salinities under 0.5–4.5 M NaCl and achieved the maximum (4.66±0.06 U/mg) at 4.5 M NaCl in continuous cultivation, then the activities decreased under 5.0 M NaCl. The backward reaction activities showed the similar trend, but the maximum (5.11±0.06 U/mg) was detected under 3.5 M NaCl, and then the activities decreased. In addition, the forward reaction activities of Dhar were lower than the backward reaction activities under 0.5–3.5 M NaCl, but were significantly higher than the backward reaction activities under 4.0–5.0 M NaCl (Figure 4). Under hypoosmotic or hyperosmotic shock, the forward reaction activities of Dhar were always lower than the backward reaction, but both had almost no correlation with the changes of salinity (Figure 4).

**Figure 5:**
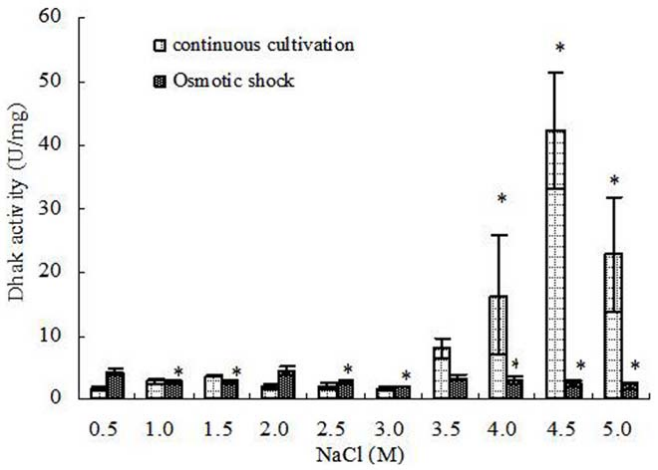
Dhak activity in *D. salina* cells under different salinity stresses. Columns represent the means of three replicated studies in each sample, with the SD of the means (T test, P<0.05). The significance of the differences between the control (2.0) and test values were tested by using one-way ANOVA. *, P<0.05 vs control.

Dhak had nearly no activity changes under continuous cultivation from 0.5 to 3.0 M NaCl (<5 U/mg), but the activities increased significantly with further increased salinities and achieved the maximum (42.19±9.14 U/mg) under 4.5 M NaCl. Under 5.0 M NaCl, Dhak was also showed a high activity of 22.85±9.05 U/mg. However, when *D. salina* treated by hypoosmotic or hyperosmotic shock, there were no significant correlation between Dhak activities and salinities, and its activities in all treatments were similar to that of cells grown continuously under 0.5–3.0 M NaCl (Figure 5).

### Relationship Between Enzyme Activity and Salinity

Statistical analysis showed that, in continuous cultivation, the activities of G3pp were significantly positively connected to the salinity (correlation test, r = 0.778>0, P<0.01) (Figure 3). Similarly, the forward reaction activities of Dhar and the activities of Dhak were also significantly positively connected to the salinity (correlation test, forward reaction activities of Dhar, r = 0.796>0, P<0.01; activities of Dhak, r = 0.827>0, P<0.01) (Figure 4, 5). However, G3pdh activity had no significant correlation with salinities (P>0.05), although its activities kept in high levels in higher salinity from 3.0 to 4.5 M NaCl (Figure 2).

Compared with the continuous salt stress, the effects of short-term salt stress on the activities of G3pdh, G3pp, Dhar and Dhak in *D. salina* were different obviously. Both the forward and backward reaction activities of G3pdh in *D. salina* treated by hypoosmotic or hyperosmotic shock were significantly positively connected to the salinity (correlation test; forward reaction activity: r = 0.674>0, P<0.05; backward reaction activity: r = 0.878>0, P<0.01) (Figure 2). On contrary, statistical analysis showed that the activities of other three enzymes had no significant correlation with salinities (P>0.05) (Figure 3, 4 and 5). Therefore, G3pdh were more sensitive to the rapidly changed salinity, which might be important in the regulation of glycerol metabolism and its flow direction under various salt stresses.

Some studies have shown similar results to this study. For example, compared with the control (no NaCl), glycerol-3-phosphate dehydrogenase (ctGPD) activity in the osmophilic yeast *Candida krusei* showed a rapid increase under hyperosmotic condition (0.34 M NaCl) [23]. However, another study showed that the DHAP reductase (G3pdh) activity in spinach (*Spinacia oleracea L.*) under 0.1 M NaCl higher than the control (no NaCl) decreased rapidly when NaCl increased from 0.1 M to 2 M NaCl [24]. In a methanol-grown yeast *Hansenula ofunaensis*, the relative activity of Dhar, compared to salt-free, decreased rapidly when NaCl concentration increased from 5 mM to 400 mM [25], which was just the opposite conclusion of this experiment (except the backward reaction activities under 4.0–5.0 M NaCl) (Figure 4). It seems that salt-tolerant species have more similar method in the regulation of enzyme activity under salt stress.

### Forward and Backward Reaction Activities of G3pdh and Dhar

(NAD^+^)-dependent G3pdh catalyzes a reversible reaction of DHAP to glycerol-3-phosphate in the pathway of glycerol synthesis. In the pathway of glycerol degradation, Dhar also catalyzes a reversible reaction between DHA and glycerol (Figure 1). Under osmotic stress, G3pdh and Dhar play important roles in glycerol accumulation or degradation in *Dunaliella* for osmotic adjustment to respect to various salinities [11], [26].

Under both continuous and shock cultivations, the forward reaction activities of G3pdh were much higher than the backward reaction activities, except in the continuous cultivation at the highest salinity 5.0 M NaCl where the activity of backward reaction was higher than that of forward reaction (Figure 2). In contrast, the forward reaction activities of Dhar were obviously lower than the backward reaction activities, except in the continuous cultivation at the highest salinities 4.0, 4.5, and 5.0 M NaCl where the activities of forward reaction were higher than backward reaction (Figure 4). Therefore, we conclude that, overall, the reversible reactions of G3pdh and Dhar tended to the direction of glycerol synthesis by regulating the enzyme activity rather than the reverse reaction to meet the required amount of glycerol. Under the highest salinities the single cell glycerol content increased rapidly [17]. With the excessive accumulation of product glycerol in cell, the reverse reactions of the two enzymes tended opposite to the direction of glycerol synthesis (Figure 1). Dhar directly catalyzes a reversible reaction between DHA and glycerol, it was more sensitive to the glycerol accumulation and began to respond at 4.0 M NaCl, while G3pdh, a enzyme indirectly catalyzes synthesis of glycerol (Figure 1), responded this at 5.0 M NaCl.

### Enzyme Activity and Glycerol Metabolic Flow Direction Under Continuous Salt Stress

2.0 M NaCl is the optimal salinity for the growth of *D. salina*, under which the activities of the four enzymes were low (Figure 2, 3, 4, 5). During continuous salt stress, the activities of the four enzymes were relatively lower under low salinities, and the changes of their activities were insignificant when salinity was lower than 2.0 M NaCl, or even lower than 3.0 M NaCl (Figure 2, 3, 4, 5), indicating that a large number of glycerol accumulation was not required in osmotic adjustment of *D. salina* under low salinity [17]. In our previous studies, the accumulation of single cell glycerol increased with increased salinity when *D. salina* was cultured chronically at various salinities, and high single cell glycerol content has been detected in cells under high salinities [17]. Under high salinities, G3pdh activities increased geometrically, and G3pp activities increased obviously with the increased salinities (Figure 2, 3) to meet the needs of cells to glycerol, which was illuminated that both enzymes play important roles in glycerol synthesis under chronic high salinities. Corresponding to this, the reverse reaction activity of Dhar to the glycerol synthesis significantly increased, and Dhak activities also increased geometrically towards the direction of the glycerol synthesis (Figure 1, 4, and 5).

Different from G3pdh and Dhak, during this process, the increase or decrease of Dhar activity was relatively flat, suggesting that Dhar played a role of balancing the cycle metabolic pathway by its forward and backward reactions to make the pathway of glycerol metabolism go on steadily.

Overall, the metabolism of intracellular glycerol in *D. salina* grown continuously under different salinities was not determined by any single enzyme but by the synergistic action of the four enzymes, which made the cell maintain lower glycerol level under low salinities and higher glycerol level under high salinities for *D. salina* to maintain osmotic balance.

### Enzyme Activity and Glycerol Metabolic Flow Direction Under Osmotic Shocks

Compared with the continuous salt stress, the short-term osmotic shocks did not result in significant enzyme activity changes. Although the trends were unconspicuous, hyperosmotic or hypoosmotic shock could clearly stimulate both forward and backward reaction activities of G3pdh (Figure 2) as indicated above. However, the activity changes of other three enzymes were minor and kept stable basically (Figure 3, 4 and 5). This rapid response to salinity suggested that the glycerol-DHAP cycle pathway was driven by G3pdh under osmotic shocks, and G3pdh was the rate-limiting enzyme in whole pathway, which regulated the glycerol level to balance the osmotic pressure caused by osmotic shock. In our previous studies, *D. salina* could rapidly decrease or increase single cell glycerol contents to adapt to hypoosmotic or hyperosmotic shock, and the accumulation of single cell glycerol also increased rapidly with increased salinity [17]. Therefore, the glycerol synthesis or degradation in *D. salina* treated by salt stress was due to the synergistic effect of four enzymes, which were propitious to the glycerol metabolism for osmotic adjustment of *D. salina* to respect to salt stress, and G3pdh play the key role in glycerol metabolism. In our previous study, it was found that several candidate motifs of the promoter of phytoene synthase (Psy), the first regulatory point in carotenogenesis in *Dunaliella bardawil*, exhibited salt-induced characteristics [27]. Psy is recognized as the rate-limiting enzyme in carotenoids synthesis. We have reason to believe that some similar motifs should exist in G3pdh, which requires research on its promoter to prove in the future.

In summary, when *D. salina* was treated by hyperosmotic or hypoosmotic shock, among G3pdh, G3pp, Dhar and Dhak, only the activity of G3pdh expressed significant correlation with salinity, suggesting that the whole cycle metabolic pathway of glycerol was driven by G3pdh when *D. salina* encountered osmotic stresses. Under continuous cultivation, lower salinity had little effect on the activity of the four enzymes, and the enzyme activity remained at a low level. When the salinity exceeded 2.0 M NaCl, optimum growth salinity of *D. salina*, the activity of the four enzymes begin to be activated in varying degrees. Under the high salinity conditions, the four enzymes were in a state of high activity to ensure the accumulation of glycerol to resist high salt stresses. Forward reaction activity of G3pdh was higher than its backward reaction, and backward reaction activity of Dhar was higher than its forward reaction, both of which were ways to make the metabolic direction to the glycerol synthesis. Lasting high salt stress resulted in the excess of single cell glycerol content, and led the reversible reactions of G3pdh and Dhar opposite to the direction of glycerol synthesis. Dhar could balance the cycle metabolic pathway by its forward and backward reactions. G3pdh is considered as a rate-limiting enzyme in the metabolic pathway, research on its gene promoter will be an interesting and worthwhile work in the future. In addition, we have examined the effect of salt upshock/downshock on the level of the mRNA coding for a (NAD^+^)-dependent G3pdh [17]. It should be more relevant to examine the effect of salt upshock/downshock on the level of the mRNA coding for the four enzymes.
