# Correction: Comparative Analysis on the Key Enzymes of the Glycerol Cycle Metabolic Pathway in *Dunaliella salina* under Osmotic Stresses

**DOI:** 10.1371/annotation/4e0ada11-9c9b-44d7-9fd0-cfe5b45c42b1

**Published:** 2012-09-12

**Authors:** Hui Chen, Yan Lu, Jian-Guo Jiang

There is an error in Figure 1. The wrong image was used. The legend is correct. The correct image for Figure 1 can be seen here: 

**Figure:**
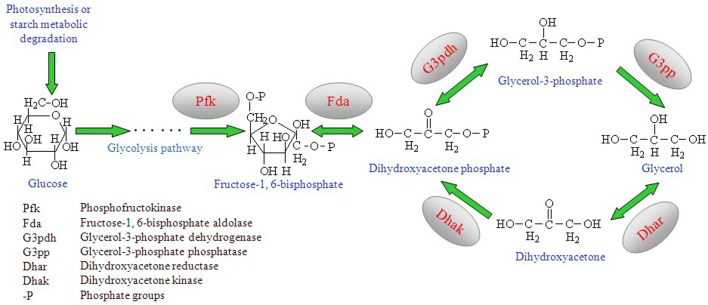


[^] 

